# Shapley-Based Estimation of Company Value—Concept, Algorithms and Parameters

**DOI:** 10.3390/e23121598

**Published:** 2021-11-28

**Authors:** Jacek Mercik, Barbara Gładysz, Izabella Stach, Jochen Staudacher

**Affiliations:** 1 Faculty of Finance and Management, WSB University in Wrocław, Fabryczna 29-31, 53-609 Wrocław, Poland; 2 Department of Operations Research and Business Intelligence, Faculty of Management, Wrocław University of Science and Technology, Wybrzeże Wyspiańskiego 27, 50-370 Wrocław, Poland; barbara.gladysz@pwr.edu.pl; 3 Department of Business Informatics and Management Engineering, Faculty of Management, AGH University of Science and Technology, Al. Mickiewicza 30, 30-059 Kraków, Poland; istach@zarz.agh.edu.pl; 4 Fakultät Informatik, Kempten University; Bahnhofstr. 61, 87435 Kempten, Germany; jochen.staudacher@hs-kempten.de

**Keywords:** company value, Shapley value, cooperative game theory, indirect personal control

## Abstract

The aim of the article is to propose a new method of valuation of a company, considering its ownership relations with other companies. For this purpose, the concept of the Shapley value from cooperative game theory is used as the basis for assessing such dependent companies. The paper presents proposals for Shapley value calculation algorithms for our model. We expand our model by discussing personal relations in addition to ownership relations and point out how intuitionistic fuzzy sets may be helpful in this context. As a result, we propose two new expanded models. In the first probabilistic model, we apply Pearson’s correlation coefficient, in the second, we use a correlation coefficient between intuitionistic fuzzy sets to determine the personal relationships. Finally, we present and interpret results for a real-world economic network with 17 companies.

## 1. Introduction

The value of a company is assessed in many ways (e.g., stock exchange value, accounting value, replacement value, etc.) depending on the needs that such an assessment serves. None of the methods takes into account the full complexity of ownership dependencies in a given company. The owners of the company may, for example, also be owners or co-owners of other business entities with or without economic relations with the analyzed company. The aim of the article is to propose a new method of valuation of a company, considering its ownership relations with other companies. For this purpose, a variant of the Shapley value [1] from cooperative game theory is used as the basis for assessing such dependent companies.

The proposed assessment of a company’s market value meets, by definition, the conditions related to the Shapley value for cooperative games. The use of the Shapley value results from its universality. It should be noted, however, that there are many different modifications to this value in the literature (e. g., [2,3,4,5,6,7,8,9,10,11,12,13,14]). Their diversity is mainly related to the diversity of assumptions concerning the coalition formation process and the assessment of the contribution of a participant in a coalition to the entire system.

If a given company is wholly owned by owners (known as shareholders, etc.) who have no other assets besides the company and does not own other assets itself (as a company), it can be assumed that the method used for valuing that company depends solely on the purpose for which this is performed, and other aspects resulting from ownership relations do not need to be taken into account. In this situation, the method of company valuation should not be changed, as it gives values acceptable to users.

The situation is different if the owners of a given company, or the company itself, are included in the ownership structure of other companies in a given market. The fact that, for example, a given company has shares of another company, which in turn has shares of a given company (or another company), has an impact on its valuation, including when, apart from ownership relations, these companies do not have anything in common and they can operate in various industries and markets. For example, the fact that a given company is co-owned by a large, listed company has a direct impact on the risk assessment related to the functioning of the audited company and thus on the assessment of the real value of the analyzed company, i.e., the market value of the first company affects the market value of the second. This relationship is reciprocal, although it does not have to be symmetrical.

Our approach to the Shapley-based estimation of a company value could be helpful in an assessment of the true value of firms. Some firms/companies hide their true values. They create a chain of firms in which one has ownership of others and register their firms in tax havens. As a result, it is difficult to assess their true values. Our model considers not only market capitalization but also network and personal connections between firms. We think that in order to estimate the value of companies closer to their true values, and thus to reveal their hidden values, ownership connections and personal connections are the two most important factors (even though we acknowledge that there might be other factors). As a tool to reveal the hidden value of a company and to make decision processes about investing more rigorous, we propose new methods based on the theory of cooperative games. Our scientific approach is deductive rather than inductive. We first derive new models for networks with ownership and personal relations based on cooperative game theory and graph theory and apply these models to real-world data in the penultimate section.

The structure of the article is as follows: in the Section 2, we discuss the assumptions describing the ownership structure in a given market. In the Section 3, we present our new method of market valuation of a given company based upon ownership structures. In the Section 4, we describe the formal properties of the approach used. In Section 5 we extend our model by incorporating personal relations and discuss an example of the proposed method on a relatively small corporate network and we present an alternative approach to the problem of personal dependencies between companies using fuzzy set theory. In Section 6, we apply our method of valuation of companies in a closed market to a real-world automotive shareholding network with personal dependencies. Finally, we present some conclusions and remarks about future research.

## 2. Preliminary Assumptions

**Figure 1:**
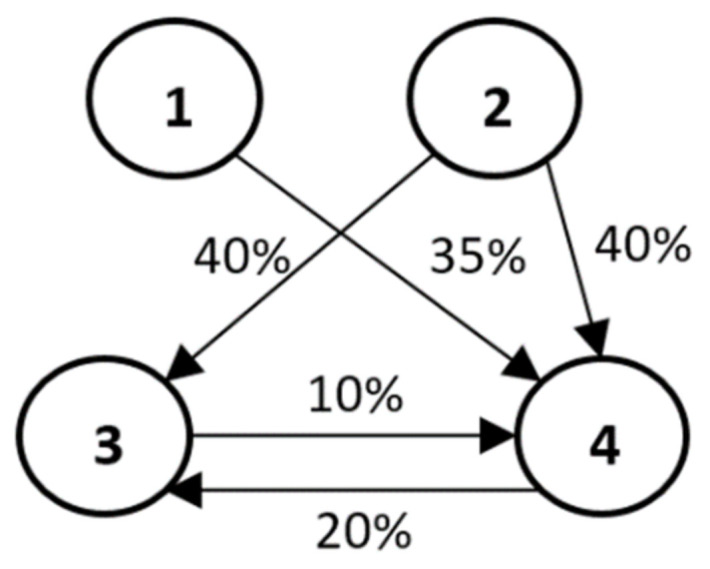
Structure of four companies with shares. Similar to network in [15].

Let the set $M=\left\{a_{i}\right\}$, i=1,2,…,n, describe a set of companies that results from an unobservable set of companies connected by ownership relations. These companies create the market of these companies, which is not necessarily homogeneous in terms of, for example, industry or organizational form (e.g., joint-stock companies, limited liability companies, etc.). In Figure 1, we present an example of such a market, consisting of four companies in different relationships with each other.

We assume that each company $a_{i}\in M$ has its value $w_{i}$. This results from the use of one method of valuation common to all $a_{i}\in M$ adequate to a given valuation task, e.g., a method of valuation through the stock exchange, accounting valuation, etc. The vector $\bar{W}=\left(w_{1},\ldots w_{n}\right),\;w_{i}\geq 0,\;i=1,\ldots ,n$, describes all companies whose relations we analyse.

The companies may hold each other’s shares, which is described by the companies share matrix $S={\left[s_{i,j}\right]}_{n,n}\;i,j=1,\ldots ,n$. We interpret the values of $s_{i,j}$ as the share of the *i*-th company in the *j*-th company. If the *i*-th company does not have any shares in other companies and no other company has shares in this company, in the sense of the share matrix *S*, then such a company is called an isolated company in the sense of the matrix *S*.

When two companies are not linked by the relationship described by the matrix *S*, but there is at least one of the natural persons (Note that non-physical owners are already included in the relationships represented by the share matrix *S*) acting as the owner or shareholder in both companies, such a relationship creates an additional type of dependency between these companies. We call it a personal dependency, $P=\left[p_{i,j}^{r}\right],i\neq j;\;i,j=1,\ldots ,n$. *r* belongs to the set of owners of $R\left(i,j\right)$. The values of $p_{i,j}^{r}$ may, for example, be the values of shares in the *i*-th company, owned by the *r*-th owner, influencing the *j*-th company (It seems that, in the most general terms, such an impact should be presented as a utility function describing the usefulness of the values of individual shares in the companies to their owners. As the shape itself and specific values of the utility function do not affect the presented method of valuation of the company, we maintain a simplified description of the personal relationship. Hence, we assume that the utility in this case is the same as the value of the share).

Note that, by our definition of $R\left(i,j\right)$, many different personal relationships can occur simultaneously between companies *i* and *j*.

Natural person business owners can therefore create an additional structure. If each such natural person is the owner solely of one company or shares in it, then we assume that this fact does not affect the market value of the other companies; we call such a company, isolated in the sense of the matrix, *P*.

The market *M* consists of companies that are not isolated in the sense of the matrix *S* or in the sense of the matrix *P* and remain in at least one of the above relationships with each other. Thus, two companies $a_{i},\;a_{j},\;i\neq j$ belong to the market *M* if they are related by their shares $s_{i,j}\;,\;s_{j,i}$ $(s_{i,j}\;,\;s_{j,i}$ are not simultaneously 0) and/or in a personal relation $P=\left[p_{i,j}\right],\;i,j=1,\ldots ,n$. In the present study, we only define the market values of companies from a given market.

A company is called an isolated company if it is isolated due to *S*-type and *P*-type relationships. Such an isolated company does, therefore, not belong to a given market *M*. If the company is not an isolated company from a given market *M*, the market value of such a company does not have to equal the value obtained after applying the selected valuation method.

Let us denote the market value of the *i*-th company as $w_{i}^{m}$.

We present the dependencies of company shares and personal relations as directed graphs $G=\langle M,\;T\rangle$. In a graph *G*, companies are represented by vertices (*M*) and the considered ownership relations (*T*) are presented as directed arcs (e.g., as displayed in Figure 1). Depending on the context, the market *M* is a market determined by the matrix *S* (market $M^{S}$), the personal relationship *P* (market $M^{P}$), or both SxP relationships (market $M^{SP}$). At the same time, dependencies *T* are dependencies of the type *S*, the type *P* or both. Note that the ownership relations (arcs) do not imply any real flows in the graph *G*, and, in this sense, the graph *G* should not be interpreted as, e.g., a transportation graph.

## 3. The Market Value of the Company with the Ownership Structure

The ownership structure is the basic relationship between companies in a given market. The assessment of a company’s value in a given market should begin with taking these relationships into account.

The matrix of participation of companies $S=\left[s_{i,j}\right]$ describes all possible connections (relations) occurring in the graph G describing the market $M^{S}$. Note that there is no logical possibility for more than two direct connections (arcs) to exist between two vertices of the graph *G*, thus the matrix *S* describes all possible situations. Moreover, we consider only connected graphs, in the sense that there are no isolated vertices (isolated companies) in them.

The matrix *S* has the following properties:

- It is not symmetrical (mutual shares do not have to be equal);
- $0\leq s_{i,j}\leq 1$ (one cannot own more than 100% of shares of a given company);
- $s_{i,i}=1\;\left(\mathrm{the}\;i-\mathrm{th}\;\mathrm{company}\;“\mathrm{owns}”\;100\%\;\mathrm{of}\;\mathrm{itself}\right)$ (Note that, for clarity and aesthetics of presentation, we do not draw any self-loops around vertices representing companies throughout the paper).

If $\sum_{l=1}^{n}s_{l,i}=\sum_{j=1}^{n}s_{i,j}=1$ then the *i*-th company is represented by an isolated vertex, i.e., the company is isolated and does not belong to a given market. In such a case, we remove the *i*-th row and *i*-th column from the matrix *S* and such a company is not analyzed. The matrix *S* with dimension *n*x*n* is transformed into a matrix with dimension $\left(n-1\right)x\left(n-1\right)$.

The value of the share of the *i*-th company in the *j*-th company is $w_{i,j}=s_{i,j}w_{j}$. Thus, the matrix $W=\left[w_{i,j}\right],\;i,j=1,\ldots ,n$ describes the values of all companies and their shares in a given market *M*.

The market value of a company isolated from the market $M^{S}$ is equal to its value determined according to the established valuation method. The value of the entire market $M^{S}$ consisting exclusively of isolated companies is equal to the sum of the values of these companies.

Companies that are not isolated companies and are connected by significant ownership relations form the so-called share structure (abbreviated as structure) described by the graph $G^{S}=\langle M^{S},S\rangle$. The value of the structure, $w\left(M^{S}\right)$, is the sum of the values of its elements (companies), i.e., $\sum_{i=1}^{n}w_{i}=\sum_{i=1}^{n}w_{i}^{m}=w\left(M^{S}\right)$. This means that the value of the entire market, estimated according to the applied valuation method, considering all possible relations between the companies, does not change, and only the proportions of the values of companies from this market resulting from taking into account these relations may change. Still, inequalities of the form $w_{i}\neq w_{i}^{m}$ for $a_{i}\in M^{S}$ are possible and subject to our investigations.

We assume, as a fact, that if the *i*-th company has shares in another company, its market value $w_{i}^{m}$ should increase (or at least not decrease). Similarly, the fact that some other company owns a stake in a company should reduce the market value of that company. Since it may be that at the same time a given company from the market $M^{S}$ has shares in another company and another company from the same market has its shares, the net result of these relationships is:

$$w_{i}^{netto}=\sum_{i=1}^{n}w_{i,j}-\sum_{l=1}^{n}w_{l,j}$$

Hence it directly follows that the sum of the net worth of companies across all companies in a market is zero:

$$\sum_{i=1}^{n}w_{i}^{netto}=\sum_{i=1}^{n}\sum_{j=1}^{n}w_{i,j}-\sum_{i=1}^{n}\sum_{l=1}^{n}w_{l,i}=0$$

If a given market $M^{S}$ can be represented as the sum of non-empty subsets of $M^{\prime }⊕\;M^{\prime \prime }$ such that $M^{S}=M^{\prime }\cup M^{\prime \prime }$ and $∄\left(p,r\right):\;s_{p,r}\neq 0,\;a_{p}\in M^{\prime },a_{r}\in M^{\prime \prime }$, then we analyze separately for each of the sets M′ and M″ and the set $M^{S}$ does not form a connected graph. This means that there is no ownership relation between the collections of the companies *M*′ and *M*″.

Let there be a connected market $M^{S}$, i.e., a market where the following conditions are simultaneously met:

There is no isolated company in it.

The companies of this market form a connected graph $G=\langle M^{S},\;S\rangle$.

The market value $w_{i}^{m}$ of the company $a_{i}$ from the market $M^{S}$ is the average value of contributions made by a given company to a given market. For this purpose, we use a method informed by the Shapley value [1,16], a well-established solution concept for cooperative games.

All companies belonging to a given market $M^{S}$ form the so-called grand coalition. Suppose this market is created in such a way that there is only one company at the beginning $a_{i_{1}}$ where $i_{1}$ is one of the numbers 1,2,…,n. It is an isolated company and thus its market value is equal to its value: $w_{i_{1}}^{m}=w_{i_{1}}$.

Then another company $a_{i_{2}},\;i_{2}\neq i_{1}$ from the market $M^{S}$ market joins. The value of the ordered coalition $\left(a_{i_{1}},a_{i_{2}}\right)\subseteq M^{S}$, i.e., the market consisting of companies $a_{i_{1}}$ and $a_{i_{2}}$, is

$$v_{\left(i_{1},i_{2}\right)}=v_{i_{1}}+w_{i_{2}}+w_{i_{2}}^{netto}=w_{i_{1}}+w_{i_{2}}+w_{i_{2}}^{netto}$$

where $v_{i_{1}}$ is the company’s value calculated using the selected valuation method (book value, replacement value, etc.).

The company $a_{i_{2}}$ makes a contribution to the ordered coalition $\left(a_{i_{1}},a_{i_{2}}\right)$ equal to

$$v_{\left(i_{1},i_{2}\right)}-v_{\left(i_{1}\right)}$$

According to the same scheme, we add another company $a_{i_{3}},i_{3}\neq i_{2}\neq i_{1}$, to the ordered coalition $\left(a_{i_{1}},a_{i_{2}}\right)$

$$v_{\left(i_{1},i_{2},i_{3}\right)}=v_{\left(i_{1},i_{2}\right)}+w_{i_{3}}+w_{i_{3}}^{netto}=w_{i_{1}}+w_{i_{2}}+w_{i_{3}}+w_{i_{2}}^{netto}+w_{i_{3}}^{netto}$$

and its contribution is accordingly

$$v_{\left(i_{1},i_{2},i_{3}\right)}-v_{\left(i_{1},i_{2}\right)}$$

We keep following this scheme until we exhaust elements of the set $M^{S}$.

Note that $\left(i_{1},i_{2},\ldots ,i_{n}\right)$ is one of the permutations of the set $\left\{1,2,\ldots ,n\right\}$. Conducting the above calculations for all permutations of the set $\left\{1,2,\ldots ,n\right\}$ and then averaging the results for each of the companies separately, we find the average contribution of a given company to the market $M^{S}$. This value is the market assessment of a given company $w_{i}^{m}$ in the market $M^{S}$.

**Example 1.** Let us consider the example of the ownership structure presented in Figure 1. This structure consists of a market of four companies {1, 2, 3, 4} with shares presented as directed arcs (*i*, *j*) of the graph, the weights of which denote the percentage of shares of individual companies (beginning of the arc at vertex *i*) in a given company (end of arc at vertex *j*).

We consider the market $M^{S}=\left\{a_{1},a_{2},a_{3},a_{4}\right\}$, in which none of the companies is isolated.

The values (e.g., stock exchange) of the companies are $\bar{W}=\left[50,25,25,30\right]$, respectively.

The individual shares of companies from the market $M^{S}$ are presented in the matrix *S*:

$$S=\left[\begin{array}{cccc} 1 & 0 & 0 & 0.35\\ 0 & 1 & 0.4 & 0.4\\ 0 & 0 & 1 & 0.1\\ 0 & 0 & 0.2 & 1 \end{array}\right]$$

Hence the values of the companies and their subsidiaries are given by the matrix $W=S\cdot {\bar{w}}^{T}$

$$W=\left[\begin{array}{cccc} 50 & 0 & 0 & 10.5\\ 0 & 25 & 10 & 12\\ 0 & 0 & 25 & 3\\ 0 & 0 & 5 & 30 \end{array}\right]$$

and we derive ${\bar{w}}^{netto}=[w_{i}^{netto}]=\left[\sum_{j=1}^{n}w_{i,j}-\sum_{l=1}^{n}w_{l,j}\right]$=$\left[10.5,\;22,-12,-20.5\right]$. Note that the sum of the net values for the entire structure is equal to 0.

Let us calculate contributions of individual companies to the grand coalition. For example, for the ordered set {1, 2, 3, 4}, we find:

$v_{\left(1\right)}=w_{1}=50$, $v_{\left(1,2\right)}=50+25+22=97$, $v_{\left(1,2,3\right)}=50+25+25+22-12=110$ and $v_{\left(1,2,3,4\right)}=50+25+25+30+22-12-20.5=119.5$.

Individual companies from the ordered set (1,2,3,4) contribute to the system, respectively:

$v_{\left(1\right)}=50$, $v_{\left(1,2\right)}-v_{\left(1\right)}=97-50=47$, $v_{\left(1,2,3\right)}-v_{\left(1,2\right)}=110-97=13$ and $v_{\left(1,2,3,4\right)}-v_{\left(1,2,3\right)}=119.5-110=9.5$.

The above calculations must be made for all 4! = 24 permutations and then averaged for the individual companies. The results of these calculations are given in Table 1.

The calculations in Table 1 show the average contribution that a given company makes to the structure $M^{S}$. The original value of the companies $\bar{w}=\left[50,\;25,\;25,\;30\right]$ transform into the market values $\bar{w}\left(M\right)=\left(57.875,\;41.500,\;16.000,\;14.625\right)$ once we include the ownership relations between these companies (Figure 1). This means that company number 1 is underestimated in average (market value) by 13% and company number 2 is underestimated by 66%. On the other hand, companies 3 and 4 have revalued values by 36% and 51.25%, respectively. When considering the purchase of shares in these companies, this should be reflected in higher price offers for companies 1 and 2 and lower ones for companies 3 and 4.

**Example 2.** Consider the companies from Example 1 (Figure 1) again, assuming this time that they have the same values resulting from the applied method of their valuation. For the sake of illustration, assume that each of the four companies has the same value of 100. Assuming that the ownership relations are still the same as in Figure 1, the Shapley values are: 126.25, 160.00, 62.50, 51.25. We therefore believe that, for companies with, for example, equal stock exchange values, the observed different Shapley values reflect not so much the market values of the companies, but the assessment of the value of the ownership relations between these companies. This time, they indicate which companies (despite equal values) dominate a given market (companies 1 and 2), which are dominated (companies 3 and 4), and to what extent. This means that, for example, when buying a 1% share in the company number 1, worth 1 unit, we actually buy 0.316% of the value of this market rather than only 0.25% (1 unit from a market worth 400 units). Of course, after the transaction is completed, we will be dealing with a new situation and thus perhaps with new market values of companies from a given market. In the extreme case, when the purchase is carried out by a company that does not belong to a given market *M*, this also changes the market *M* itself.

Moreover, relations (ownership shares) within a given market *M* can be selected in such a way as to maximize the Shapley value for a given company or increase its profit. For example, company 1, by increasing its shares (ceteris paribus) in company 4, increases its market value from 126.25 to 137.50, that is by 11.25 units at the investment cost of 15 units. In this case, it seems, investing in company 4 has no economic justification and no further investment can take place (the sum of shares in company 4 cannot exceed 100%). Moreover, the reduction of shares (below 0.35) of company 1 in company 4 obviously lowers the market value of company 1. For example, a reduction of shares of company 1 in company 4 from 0.35 to 0.30 lowers the market value of company 1 from 126.25 to 122.5, i.e., by 3.75 units; however, it results in a profit of 5 units, which may be deliberate (Of course, in the case of purchases made by companies not belonging to the market M so far, it is necessary to analyse the modified market by adding these companies).

**Example 3.** Again, we consider the situation presented in Figure 1. Let us assume, as before, that the percentages on the arcs describe the ownership shares of individual companies. The values assigned to the vertices, on the other hand, describe the market share of a given company and assume that this market is uniform in terms of the area in which these companies operate (e.g., they are companies exclusively from the consumer electronics market or only companies from the area of tourist services, etc.). The Shapley value estimates of the market value of such firms correspond to the estimates of the market share of these firms. For example, assuming (ceteris paribus) that these companies have nominally equal market shares (25% each), we find that ownership relationships (such as in Figure 1) change these proportions and imply the actual market shares equal to 0.30625, 0.40000, are, respectively, 0.15625, 0.13750. The obtained results indicate that, in this case, investing in companies 1 and 2 means investing in companies that have a greater than nominal market share, i.e., that such a procedure may have higher economic efficiency than is apparent from the nominal value of companies from a given market.

The model presented in Section 3 of our paper uses a permutation-based approach which is very strongly related to the computation of the Shapley value. In the following, we point out that, in our case, there is a closed formula and direct algorithm for the computation of our permutation-based value for n companies based on the vector

$$\bar{w}=\left[w_{1},\ldots ,w_{n}\right]$$

of the original company values and the vector

$${\bar{w}}^{netto}=\left[w_{1}^{netto},\ldots ,w_{n}^{netto}\right]$$

derived from the ownership structure. According to our model, the marginal contribution of the first company $i_{1}$ in every permutation is $w_{i_{1}}$, because we view $i_{1}$ as an isolated company. The marginal contribution of any other company $i_{j},j=2,\ldots ,n,$ is

$$w_{i_{j}}+w_{i_{j}}^{netto}.$$

Noting that every company occupies the first spot in a permutation with equal frequency $\frac{1}{n}$, we can find a closed formula for our Shapley values (In the vein of generalised Shapley value [11] as we point out in Section 4) of company i.

(1) $$Sh\left(i\right)=\frac{1}{n}\cdot w_{i}+\frac{n-1}{n}\cdot \left(w_{i}+w_{i}^{netto}\right)=w_{i}+\frac{n-1}{n}\cdot w_{i}^{netto}$$

Note that for n→∞ there holds $Sh\left(i\right)\to w_{i}+w_{i}^{netto}$. Therefore, the value $w_{i}^{netto}$ is crucial for “big” markets.

We acknowledge that game theoretic approaches for determining the market value of a company differing from ours are possible. We briefly sketch such an alternative approach in the following. Let us start with an isolated company $a_{i_{1}}$ and another company $a_{i_{2}}$, $i_{2}\neq i_{1}$, from the market $M^{S}$ joining it. One could simply work on the directed subgraph consisting of $a_{i_{1}}$ and $a_{i_{2}}$ and the corresponding submatrix of S. Once another company $a_{i_{3}}$, $i_{3}\neq i_{2}\neq i_{1}$, joins the coalition $\left(a_{i_{1}},a_{i_{2}}\right)$, one could simply work out the marginal contribution of company $a_{i_{3}}$ based upon the directed subgraph consisting of $a_{i_{1}}$, $a_{i_{2}}$ and $a_{i_{3}}$ and the corresponding submatrix of S. In such an alternative model, in which marginal contributions depend only on the companies that have entered the market so far, the (generalized) Shapley value $\bar{Sh\left(i\right)}$ of company i becomes

$$\bar{Sh\left(i\right)}=\frac{1}{2}\cdot w_{i}+\frac{1}{2}\cdot \left(w_{i}+w_{i}^{netto}\right)=w_{i}+\frac{1}{2}\cdot w_{i}^{netto}.$$

The above formula reflects the fact that in half of all permutations company i enters the market before company j whereas for the rest of the permutations company i enters the market after company j. Hence ownership relations between companies i and j would only be reflected half of the time.

In general, the approach presented in this paper is related to the goal of taking into account the maximum impact of the entire market (or at least parts of it as big as possible) on the value of a given element, i.e., a company. We believe that a given vertex has value and relations and enters a coalition together with its relations described by its net worth. The influence of the entire market on a given company appears unnecessarily limited in cases where marginal contributions depend only upon the companies that have entered the market so far and that is the reason why we argue for our approach, as compared to, e.g., the possible alternative model outlined in the previous paragraph.

## 4. Properties of the Proposed Market Assessment of a Given Company on the *M^S^* Market

The proposed assessment of the market value of a company, in principle, meets, by its structure, the conditions related to the Shapley value. The wide usage of the Shapley value stems from its universality, however, there are many different modifications to this value. Their diversity is related primarily to the diversity of assumptions related to the coalition formation process (in our case, the market) and the assessment of the contribution of a participant in a coalition (in our case, a company) to the entire system (market). Two measures from the set of solution concepts are assumed to play a special role: the Shapley value (Also known as the Shapley–Shubik index [16] in the case of simple games) and the Banzhaf value (The so-called the Banzhaf index [3] should be called the Penrose–Banzhaf index, as it was Penrose [17] who first gave its definition. The Banzhaf index was later generalised to general non-simple cooperative games by Owen [18]) (which is widely used as a power index for simple games, i.e., cooperative games where coalitions may only have values 0 and 1). In our opinion, Shapley’s approach is a better approach in relation to the assessment of the company’s market value, which we will show by analyzing the assumptions related to such an assessment. In the following, we provide a little theoretical background on cooperative game theory and point out why the solution concept presented in Section 3 coincides with the generalized Shapley value by Nowak and Radzik [11] for a generalized cooperative game.

A cooperative n-person game with transferable utility (TU game) is defined by a set of n players $N=\left\{1,\ldots ,n\right\}$ and a characteristic function $v:2^{N}\to \mathbb{R}$ assigning each subset $S\in 2^{N}$ a real value with $v\left(\emptyset \right)=0$. N is called the grand coalition and $\left|S\right|$ denotes the cardinality of the set S. For so-called simple games, the characteristic function v takes only the values 0 or 1, i.e., $v:2^{N}\to \left\{0,1\right\}$. A solution concept (or game value) f is a function mapping a unique vector $f\left(v\right)=\left(f_{1}\left(v\right),\ldots ,f_{n}\left(v\right)\right)$ to a given TU game specified by the player set N and the characteristic function v. If our TU game is simple, the game value f is frequently referred to as a power index (An elementary introduction to the power indices methodology can be found in [19]. Holubiec and Mercik [20] provide a variety of applications of power indices to the analysis of political processes. Bergström and Rydquist [21] analyse decision-making power in corporations. In their pioneering monograph, Felsenthal and Machover [22] present the most comprehensive survey of results of voting power analysis).

In our model, the order in which players, i.e., companies, enter a coalition is crucial. Let $\prod \left(S\right)$ denote the set of all orders of players in the set S. We call an element of $\prod \left(S\right)$ an ordered coalition. A game in generalised characteristic function form assigns a real value $v\left(T\right)$ to every ordered coalition $T\in \prod \left(S\right)$, S⊆N, with $v\left(\emptyset \right)=0$, in particular. Nowak and Radzik [11] define

$$GSh\left(v,i\right)=\underset{S\in N\setminus \left\{i\right\}}{\sum }\underset{T\in \prod \left(S\right)}{\sum }\frac{\left(n-\left|T\right|-1\right)!}{n!}\left(v\left(T,i\right)-v\left(T\right)\right)$$

as the generalized Shapley value. As pointed out [11], the generalized Shapley value (defined for the class of generalized cooperative games C) coincides with the classical Shapley value when restricted to the class of classical TU games. In the following we equate the set of players N with our set of companies M and the game values $f\left(v\right)=\left(f_{1}\left(v\right),\ldots ,f_{n}\left(v\right)\right)$ with the market values of companies $w^{m}\left(v\right)=\left(w_{1}^{m}\left(v\right),\ldots ,w_{n}^{m}\left(v\right)\right)$.

According to the definition, the set $M=\left\{a_{i}\right\},\;i=1,2,\ldots ,n,$ describes a finite set of companies present in a given market and connected by ownership relations. The value of the structure, $w\left(M\right)$, is the sum of the values of its elements (companies), i.e., $\sum_{i=1}^{n}w_{i}=w\left(M\right)$, where $w_{i}$ is the value of the company determined using a method that is established and uniform (Because the structure is an isolated one, this can be framed as, “the law of conservation of value”. This law means that at the given moment the value of the market can neither be created nor destroyed; rather, it can only be transformed or transferred from one form to another) for all companies.

Let us now define the form of the characteristic function v for the generalised cooperative game $C=\langle M,v\rangle$ describing the market $M=\left\{a_{i}\right\},$ i=1,2,…,n, and its n companies related by ownership relations.

We work with the vector $\bar{w}=\left(w_{1},\ldots ,w_{n}\right)$ of the original company values and the vector ${\bar{w}}^{netto}=\left(w_{1}^{netto},\ldots ,w_{n}^{netto}\right)$ derived from the ownership structure. Our generalised characteristic function in Section 3 has the following form: $v\left(\emptyset \right)=0$, $v\left(i\right)=w_{i}$ for all isolated companies i=1,…,n. For all orderings of companies $i_{1},i_{2},\ldots ,i_{k}$ with k=2,…,n, there holds

$$v\left(i_{1},i_{2},\ldots ,i_{k}\right)=w_{i_{1}}+\overset{k}{\underset{j=2}{\sum }}(w_{i_{j}}+w_{i_{j}}^{netto})$$

We note that the function specific to each individual company $a_{i}\in M,\;i=1,\ldots ,n$, is $v\left(a_{i}\right)=w_{i}$. Since in some methods of valuation of the company’s value (e.g., in the accounting method), this value may also be a negative value, then $v\left(a_{i}\right)\in R$. However, the market value of the company (unlike the book value) cannot, by definition, be a negative value (in the worst case it is equal to zero), then we will scale the weights so that the obtained values of $w_{i}$ are always non-negative. Let $w_{i}^{\prime }=w_{i}+\left|\min\left\{w_{1},\ldots ,w_{n}\right\}\right|$. Hence $w_{i}^{\prime }\geq 0$. As it is a reversible formal procedure, in the following considerations we will, for the sake of simplicity, use the symbols $w_{i}$, remembering that it is possible to scale if necessary.

We can easily convince ourselves that our generalized characteristic function v is monotonic, i.e., if for two ordered coalitions S and T there holds S⊆T, then $v\left(S\right)\leq v\left(T\right)$ follows.

Let us start with the ordered coalition $T=\left(a_{i_{1}},a_{i_{2}}\right)$. We are to show that for the coalition $\left(a_{i_{1}}\right)=S\subset T$ there holds $v\left(S\right)\leq v\left(T\right)$. This means $v\left(a_{i_{1}}\right)\leq v\left(a_{i_{1}},a_{i_{2}}\right)$ and the condition $w_{i_{1}}\leq w_{i_{1}}+w_{i_{2}}+w_{i_{2}}^{netto}\Rightarrow 0\leq w_{i_{2}}+w_{i_{2}}^{netto}$ must be met.

For player *i*, the net value $w_{i}^{netto}=\sum_{j=1}^{n}w_{i,j}-\sum_{l=1}^{n}w_{l,i}$ consists of two components, the first of which $\sum_{j=1}^{n}w_{i,j}$ has a minimum value of 0 (this means that the company does not have any shares in other companies) and the second has a maximum value equal to $w_{i}$ (other companies may not have more shares in company *i* than its value). Therefore, the minimal value of $w_{i}^{netto}$ is equal $\min\left(w_{i}^{netto}\right)=0-w_{i}=-w_{i}$. Hence, $\min\left(w_{i}+w_{i}^{netto}\right)=0$. The condition $0\leq w_{i}+w_{i}^{netto}$ is met.

Due to the lack of symmetry of the function $v\left(a_{i_{1}},a_{i_{2}}\right)$, we carry out similar considerations for $v\left(a_{i_{2}},a_{i_{1}}\right)$ with a similar result. By induction we can show similar properties for a larger number of players, and thus prove that our generalized characteristic function v satisfies the monotonicity condition.

As pointed out at the end of Section 3, Formula (1) is the generalized Shapley value of our generalized cooperative game v. The generalised Shapley value is the unique solution concept $f:C\to {\mathbb{R}}^{n}$ on the set of generalised cooperative games with n players satisfying the following three axioms (which we will in the following interpret for our specific application) [11].

The efficiency axioms states

$$\underset{i\in M}{\sum }f_{i}\left(v\right)=\frac{1}{n!}\underset{T\in \prod \left(M\right)}{\sum }v\left(T\right)=w\left(M\right),$$

i.e., the axiom guarantees that the sum of market values $w_{i}^{m}\left(v\right)=f_{i}\left(v\right)$ always equals the value of the market $w\left(M\right)$. All orderings of the grand coalition M are equally likely and their average is $w\left(M\right)$.

We call player i a null player in the game v if for every ordered coalition $T=\left(i_{1},i_{2},\ldots ,i_{k}\right)$ with i∉T, there holds $v\left(\left(T,i\right)\right)=v\left(T\right)$ where $\left(T,i\right)=\left(i_{1},i_{2},\ldots ,i_{k},i\right)$. The null player axiom states $f_{i}\left(v\right)=0$ for every null player i in the game v∈C. In our model, null players are companies with value 0 that do not hold any shares in companies with values greater than 0.

The third axiom is additivity. For every two games v,w∈C there holds $f_{i}\left(v+w\right)=f_{i}\left(v\right)+f_{i}\left(w\right)$. As for our application, additivity implies that we could analyze a market M with two different games v and w corresponding to two different vectors of initial company values. If we re-model the setting as a single game by adding the two vectors of company values, then the market values of all n companies would be the sums of the market values achieved under the two separate games.

As pointed out [11], the generalized Shapley value could alternatively be characterized as the unique solution concept for the class of generalized cooperative games satisfying the efficiency axiom together with the marginal contribution and the null game axioms (For similar modifications of the set of axioms for the Shapley value for classical TU games, we refer to [23], or the introduction of the concept of Young’s marginality [24]).

The marginal contributions axiom states that for every two games v,w∈C there holds $f_{i}\left(v\right)=f_{i}\left(w\right)$ whenever $v\left(\left(T,i\right)\right)-v\left(T\right)=w\left(\left(T,i\right)\right)-w\left(T\right)$ for every ordered coalition $T=\left(i_{1},i_{2},\ldots ,i_{k}\right)$ with i∉T and $\left(T,i\right)=\left(i_{1},i_{2},\ldots ,i_{k},i\right)$. The relation to Formula (1) is obvious. The way we define the marginal contribution of a company entering a market is crucial for our model.

The null game axiom states that for every null game $v_{0}$, i.e., for every generalized cooperative game $v_{0}$ that assigns the value 0 to every ordered coalition in C, there holds $f_{i}\left(v_{0}\right)=0$ for all i∈N. For our application, the null game refers to a trivial and hypothetical situation where all companies in the market M as well as their shares have value 0.

Note that the model we propose satisfies the following symmetry property: denote by $\prod \left(M\right)$ the set of all permutations of M (i.e., bijections π:M→M). For $\pi \in \prod \left(M\right)$ and a generalised cooperative game v∈C, define πv∈C by $\left(\pi v\right)\left(T\right)=v\left(\pi \left(T\right)\right)$ for all $T\in \prod \left(S\right)$, $S\in 2^{M}$. The game πv is the same as v except that the players are relabeled according to π. (Equivalent to the symmetry axiom is the so-called equal treatment axiom: If i,j∈N are substitute players in the game v∈C, i.e., for every ordered coalition S⊂M\{i,j|: v(S,i)=v(S,j)}, then $w_{i}^{m}(v)=w_{j}^{m}(v)$. According to the symmetry property, if players are relabeled in a game, their a priori market value will be relabeled accordingly. Thus, irrelevant characteristics of the players, outside of their role in the game v, have no influence on the market value.

Finally, our model exhibits a gain-loss property in the following sense: If $w_{i}^{m}\left(v\right)>w_{i}^{m}\left(w\right)$ for some v,w∈C and i∈M, then there exists j∈M such that $w_{j}^{m}\left(v\right)<w_{j}^{m}\left(w\right)$. This property captures what could be expected intuitively from a measure of power. While it might be the case that the value of some players increases as a result of changes in the game, power cannot concomitantly increase for all players. That is, any gain in value by a player must come at the expense of someone else. For our model, the gain-loss property is an immediate consequence of our law of conservation of value in the sense that the value of our market *M* is fixed at *W*(*M*).

## 5. Market Value of the Company on the Market *M^P^*—A Game Model

In the context of the assessment of the company’s market value there is a second group of relations between companies in a given market, i.e., personal relations. If these relationships are equivalent to the ownership relations investigated in the previous sections, then we could use the same approach as for ownership relations. In such instances, it is possible to separately assess the market value of a given company in terms of ownership and personal relations, leaving the evaluator to interpret the results obtained, which will almost certainly be different (Of course, it is also possible to use by analogy some other form of relationship connecting companies from a given market. It depends on the purposes for which such an analysis is performed). The assessment of the value of a given company in terms of both types of relationships requires, in these instances, some form of aggregation and a large dose of arbitrariness (e.g., using a weighted average).

In this section, we propose two new models for assessing the company’s value on the market $M^{P}$. In the first probabilistic model, we apply Pearson’s correlation coefficient, in the second, we use a correlation coefficient between intuitionistic fuzzy sets to determine the personal relationships.

### 5.1. Market Value of the Company on the Market $M^{P}$—A Probabilistic Game Model

Now, consider the market $M^{P}$ gathering companies according to the personal relations described by the matrix $P=\left\{p_{i,j}\right\},\;i,j=1,\ldots ,n$. Note that:

The collections of companies represented by the markets $M^{S}$ and $M^{P}$ do not have to coincide.

The connectivity of the graph $G^{S}=\langle M^{S},\;S\rangle$ does not mean the automatic consistency of the graph $G^{P}=\langle M^{P},P\rangle$.

In the graph $G^{P}$, multiple and different relations between companies belonging to the set $M^{P}$ (which is consistent in the sense of the relation *P*) are allowed.

The basic problem with the analysis of relationships (which we call personal relationships here) is that their value cannot be “evaluated” using measures that were used to measure the value of a given company (We remind the reader that these may be different measures for different valuation methods, but the same for a given market with ownership and personal relations). For example, the fact that the president of one company is on the supervisory board of another indicates that the policies of both companies may be somewhat coordinated. It seems that this has an impact on their ownership relations, although it cannot be determined in advance whether this influence strengthens or weakens the valuation of each of them. One can imagine a situation where the same person in one company will seek to increase the value of the company, while in the other company that person will behave in the opposite way. We are dealing with possible strategic behaviors that are impossible to capture at the time of the market valuation of the companies. Hence, if a personal relationship between two companies is to affect their policies, its existence can be described by giving the absolute value of the correlation coefficient. We call this coefficient the personal relation coefficient.

Let $r_{i,j}$ be the Pearson correlation coefficient between *i* and *j* resulting from the existence of personal relationships between companies *i* and *j*. Its absolute value will be the ratio of personal relations we are looking for. In the classic approach to examining the significance of the correlation coefficient (e.g., using the Student’s *t*-test) for a given level of significance and known degrees of freedom, we can determine the critical value, deciding whether a given level of personal relationships is sufficient to recognize them as having an impact on the market valuation of a set of companies.

Let us assume that the measure of personal relations, $r_{i,j}^{p}$, for each of two companies *i* and *j* from the market *M^S^* (where such a relation exists) is the minimal solution of the equation $t_{critical}=\frac{r_{i,j}^{p}\sqrt{N-2}}{\sqrt{1-r_{i,j}^{p^{2}}}}$, where *N* is the number of companies analyzed on the *M^S^* market and $t_{critical}$ is the value of the *t*-Student distribution for *N*−2 degrees of freedom and the $1-\frac{\alpha }{2}$ quantile for the α=0.05 significance coefficient.

We note that the so-defined ratio of personal relations is common for all companies from the market *M^S^* for which personal relations take place. Of course, once we have the appropriate statistical material, the values of these coefficients of personal relations can be individualized.

Summarizing, the matrix $P=\left\{p_{i,j}\right\},\;i,j=1,\ldots ,n$ is a matrix whose elements equal $p_{i,j}=1$ if there are personal relations between companies *i* and *j* and $p_{i,j}=0$ otherwise.

The matrix $P=\left\{p_{i,j}\right\},\;i,j=1,\ldots ,n$ transforms into the matrix $R=\left\{r_{i,j}\right\}$ i,j=1,…,n, where: $r_{i,j}=0\;if\;p_{i,j}=0$; $r_{i,j}=1\;if\;i=j$ and $r_{i,j}=r_{i,j}^{p}\;\mathrm{otherwise}$.

Moreover, personal relationships do not affect the ownership structure. If a given company has shares in another company, the quantity of shares is not affected, but the value of these shares may change. For each pair of the two companies from the market *M^S^*, two options should be considered:

(1) The ownership structure allows for a relationship between these companies.
(2) The ownership structure does not allow for any relationship between these companies.

Let us return to Example 1, shown in Figure 1. Let us assume the existence of a personal relationship between firm 2 and firm 4 coincides with the ownership relationship in which firm 2 owns 40% of firm 4—this is the first case mentioned above. Alternatively, the possible personal relationship between companies 1 and 2 is not reflected in the ownership structure of this market—this is the second case.

In the first case, including personal relations means the formation of a pre-coalition. For Example 1, the existence of a personal relationship between companies 2 and 4 means that we deal with the following set of players: {1, (2, 4), 3} where the pre-coalition (2, 4) should be treated as possible and a chance for its existence is dependent on the coefficient of personal relationships.

**Example 4.** Let us continue Example 1. For the market $M^{S}$ we have $\bar{w}\left(M\right)=\left[57.875,\;41.500,\;16.000,\;14.625\right]$. We have three people in this market, who we find in more than one company management: $p_{1}^{1},p_{4}^{1}$ (in company number 1 and number 4), $p_{1}^{2},p_{3}^{2}$ (in company number 1 and number 3), $p_{2}^{3},p_{4}^{3}$ (in the company number 2 and number 4).

**Figure 2:**
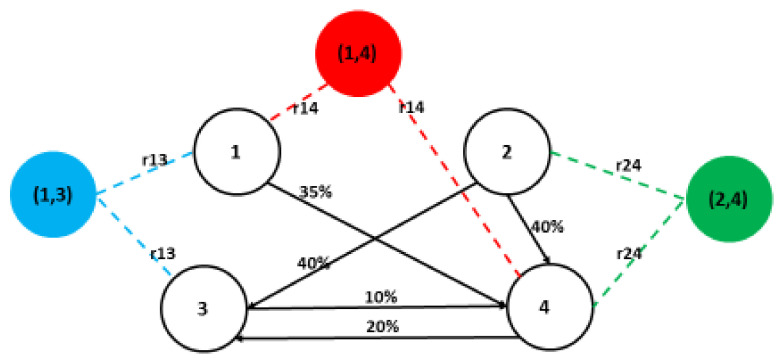
Structure of four companies with shares and personal relations ($p_{1}^{1}=p_{4}^{1}=r14$, $p_{1}^{2}=p_{3}^{2}$ = *r*13, $p_{2}^{3}=p_{4}^{3}=r24$).

Let us modify the graph in Figure 1. We added artificial nodes, that represent so-called coalitions $\left(i_{1},i_{2}\right)$, and arcs $\left(i_{1},\left(i_{1},i_{2}\right)\right)$, $\left(i_{2},\left(i_{1},i_{2}\right)\right)$ that represent measures of personal relationship for companies $i_{1},i_{2}$ where the person relationship exist, see Figure 2:

Let us assume that companies with the same people form the so-called pre-coalitions. So, we have a new market *M*′ consisting of companies and their pre-coalitions: $\left\{1,\;2,\;3,\;4,\;\left(1,3\right),\left(1,4\right),\;\left(2,4\right)\right\}$.

Suppose further that the market values of each pre-coalition are equal to the sums of the market values resulting from the ownership relationships of each of the companies in that pre-coalition. For the market *M^S^*, we obtain the following vector

$$\bar{w}\left(M^{\prime }\right)=\left[57.875,\;41.500,\;16.000,\;14.625,\;73.875,\;72.5000,\;56.125\right].$$

Of course, personal relationships do not in themselves increase the value of the market, but only affect the value relationships between companies in that market. Therefore, we standardize the values of this market amounting to 130:

$$\bar{w}\left(M_{stand}^{\prime }\right)=\left[22.62782,\;16.22556,\;6.255639,\;5.718045,\;28.88346,\;28.34586,\;21.94361\right].$$

Knowing the matrix of net values and using Formula (1), one can derive the mean values of each player for this game.

As we do not know the nature of personal relations between individual companies (we only know that such relations exist), we assume that the final value of a given company consists of the “clean” parts (resulting directly from ownership relations) and “biased” parts (resulting from participation in the pre-coalition). Of course, if we know the real proportions between “clean” and “biased” parts in the pre-coalition, then such a division should be consistent with these proportions and assigned to these companies from this pre-coalition. Since we do not know these relations in the analyzed example, we may assume that they equal 50 percent of the values of the pre-coalition for each company.

We also assume that shares of a pre-coalition in each single company (and vice versa) equal absolute values of $r_{i,j}^{p}$. For *N* = 7, α=0.05 and $t_{critical}=$ 2.571, $r_{i,j}^{p}=0.75455$ (this is the lowest estimation as we do not know what those relations look like in reality (Note that as the number of companies in the market *M* increases, the value of $r_{ij}^{p}$ decreases. This corresponds to the intuition that the impact of a specific person on the values of all companies is the smaller the more companies there are in a given market)).

Therefore, the following matrices S, W (Table 2) and net values vector $\bar{w}$ are obtained.

$$\begin{array}{ll} \bar{{\bar{w}}^{netto}}=[w_{i}^{netto}] & =\left[\overset{n}{\underset{j=1}{\sum }}w_{i,j}-\overset{n}{\underset{l=1}{\sum }}w_{l,j}\right]\\ & =\left[-5.13098\;\;8.617359\;\;11.96637\;\;22.40076-19.3357-18.9758\;\;0.458034\right] \end{array}$$

For the market $M^{S,P}$ we obtain the following values for companies 1, 2, 3 and 4: 42.195, 24.905, 23.265 and 36.635.

### 5.2. Market Value of the Company on the Market $M^{P}$—A Fuzzy Intuitionistic Game Model

The use of a generalized approach to the assessment of personal relationships based on the estimation of the correlation coefficient suffers from a significant deficit and motivates search for another method of this assessment, in which expert assessment plays a leading role. Specifically, a fuzzy approach seems appropriate.

In this paragraph we apply fuzzy theory to analyze the market $M^{P}$. First, we present the basic notion of intuitionistic fuzzy set theory. An intuitionistic fuzzy set is a fuzzy set A in a space U such that

$$A=\left\{\left(x,\;\mu_{A}\left(x\right),\nu_{A}\left(x\right)\right);x\epsilon U\right\}$$

with

$$\mu_{A}:U\to \left[0,1\right]$$

and $\nu_{A}:U\;\left[0,1\right]$.

$\;\mu_{A}\left(x\right)$ specifies the possibility that x belongs to set A, whereas $\nu_{A}\left(x\right)$ specifies the possibility that x does not belong to set A. Moreover, $\;\mu_{A}\left(x\right)+\nu_{A}\left(x\right)\leq 1$, see [25,26].

The pair $\langle \mu_{A}\left(x\right),\nu_{A}\left(x\right)\rangle$ is called the intuitionistic fuzzy value of element x in set A. The value $\pi_{A}\left(x\right)=1-\mu_{A}\left(x\right)-\nu_{A}\left(x\right)$ is called the degree of non-determinacy or uncertainty of the element xϵU in the intuitionistic fuzzy set A. This value is also called the hesitation value of x. The concept of a correlation coefficient between intuitionistic fuzzy sets A and B was first introduced in [27]. There, the following formula was proposed:

(2) $$\rho \left(A,B\right)=\frac{C\left(A,B\right)}{\sqrt{T\left(A\right)T\left(B\right)}}\;$$

where:

$$C\left(A,B\right)=\sum_{i=1}^{n}\left(\mu_{A}\left(x_{i}\right)\mu_{B}\left(x_{i}\right)+\nu_{A}\left(x_{i}\right)\nu_{B}\left(x_{i}\right)\right)$$

$$T\left(A\right)=\sum_{i=1}^{n}\left(\mu_{A}^{2}\left(x_{i}\right)+\nu_{A}^{2}\left(x_{i}\right)\right)$$

$$T\left(B\right)=\sum_{i=1}^{n}\left(\mu_{B}^{2}\left(x_{i}\right)+\nu_{B}^{2}\left(x_{i}\right)\right)$$

The correlation coefficient [27] takes values in the interval [0, 1]. Other approaches for measuring correlations between intuitionistic fuzzy sets were introduced in [28,29,30].

Let us now consider the personal relations in a market. Let us assume that we have people in the market who we find on more than one company supervisory board $P_{1},P_{2},\ldots P_{K}$. Let us assume further that companies with the same people form the so-called pre-coalitions. So, we have a new *M*′ market consisting of companies and their pre-coalitions k=1,…,K.

Let us now assume that the $P_{k}$-th person’s activity in the $i_{j}$-th company in the k-th pre-coalition $\left(i_{1},i_{2},\ldots ,i_{k}\right)$ is described by an intuitionistic fuzzy set $E_{i_{j}}^{k}$= ($\mu_{i_{j}}^{k},\nu_{i_{j}}^{k})$, $i_{j}$=$i_{1},i_{2},\ldots ,i_{k}$. Let $\mu_{i_{j}}^{k}$ specify the possibility that decisions are made by the person(s) in line with company policy (which will seek to increase the value of the company). Whereas $\nu_{i_{j}}^{k}$ specifies the possibility that decisions made by the person are not in line with company policy (thus seeking to decrease the value of a company). The values $\mu_{i_{j}}^{k},\nu_{i_{j}}^{k}$ are determined by experts based on the assessment of the person’s behavior.

**Example 5.** Consider once again the situation presented in Example 4. Let us find the values of companies considering the ownership structure along with the personal structure as in Section 5, but this time employing a fuzzy intuitionistic game model.

Taking into account the ownership structure, the standardized values of companies 1, 2, 3, 4 and pre-coalitions (1,3), (1,4) and (2,4) are equal to

$$\bar{w}\left(M_{stand}^{\prime }\right)=\left[22.62782,\;16.22556,\;6.255639,\;5.718045,\;28.88346,\;28.34586,\;21.94361\right]\;$$

The parameters of the intuitionistic fuzzy sets $E_{i_{j}}^{k}$= ($\mu_{i_{j}}^{k},\nu_{i_{j}}^{k})$ for the personal relationships in pre-coalitions (1,3), (1,4) and (2,4) estimated by experts are given in Table 3. For example, person 2 sitting on the board of company 1 and company 3 in precoalition (1,3) tends to act in favor (according to company policy) of company 1 to the degree $\mu_{1}^{2}=$ 0.7 and tends to act to the disadvantage company 1 to the degree $\nu_{1}^{2}=$ 0.25. Therefore, the uncertainty of this person’s activity on the management board of company 1 is equal to $\pi_{1}^{2}=1-0.7-0.25=0.05$. On the other hand, the evaluation of the person’s activity for benefit and disadvantage of company 3 is the same $\mu_{3}^{2}=\nu_{3}^{2}=$ 0.3 and thus the uncertainty of her activity is equal to $\pi_{3}^{2}=$ 1−0.3−0.3=0.4.

Using Formula (2) we can calculate the correlation coefficient for personal relationship in precoalition (1,3):

$$\rho \left(1,3\right)=\frac{0.7\cdot 0.3+0.25\cdot 0.3}{\sqrt{\left(0.7^{2}+0.25^{2}\right)\left(0.3^{2}+0.3^{2}\right)}}=0.90375$$

The measures of personal relationships for all pre-coalitions are given in Table 4.

Hence, the matrices *S*, *W* (Table 5) and the vector $\bar{w}$ of net values take the following forms:




${\bar{w}}^{netto}=[w_{i}^{netto}]=$

[−4.5953313.2446321.061922.9189728.1088327.7830421.47796]


Finally, we make the same assumption as in Example 4 for the nature of personal relations between individual companies, i.e., they equal 50 percent of the values of the pre-coalition for each company. For companies 1, 2, 3 and 4, their values in line with the fuzzy valuation method (incorporating both ownership and personal relations) are presented in Table 6.

We can observe that in the fuzzy model company 4 has the highest value, while in the remaining cases company 1 has the highest value. This is due, among other things, to the assumption made in this example that company 4 is part of two pre-coalitions (1,4) and (2,4). In both these pre-coalitions, persons 1 and 3 sitting on the supervisory boards of companies of pre-coalitions act much more in favor of company 4, respectively ($\mu_{4}^{1}=$ 0.8 and $\mu_{4}^{3}=$ 0.9) than in favor of the other companies in these pre-coalitions.

## 6. The Real-World Example

In this section, we provide a real-world example of a shareholding network with personal dependencies in order to test our approach.

Stellantis N.V. is a multinational automotive manufacturing corporation, formed in 2021 as a merger between the Italian–American conglomerate Fiat Chrysler Automobiles and the French PSA Group. In 2021, Stellantis is the sixth-largest automaker worldwide which is listed on Milan’s Borsa Italiana, on Euronext Paris, and on the New York Stock Exchange (see https://en.wikipedia.org/wiki/Stellantis, accessed on 12 August 2021).

**Figure 3:**
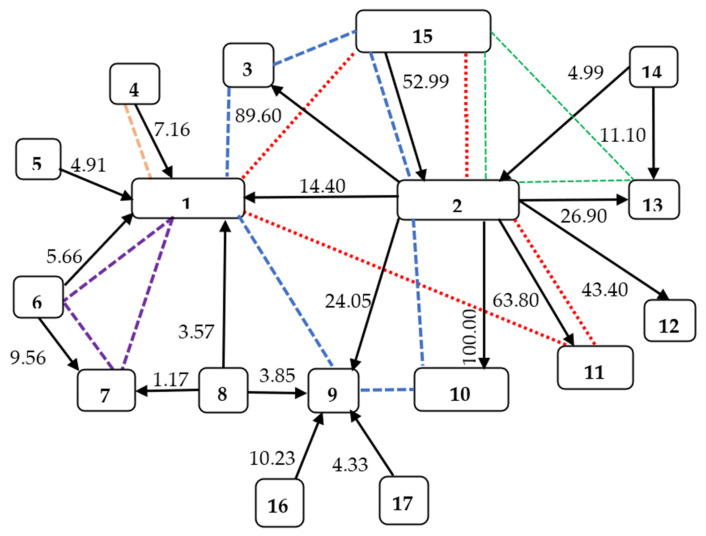
Stellantis shareholding network with shares and personal relations. 1—STELLANTIS, 2—EXOR N.V., 3—GEDI, 4—Peugeot family group, 5—Dongfeng Motor Corporation, 6—Bpifrance, 7—Orange, 8—BlackRock, 9—Ferrari, 10—PartnerRe, 11—Juventus Football Club, 12—The Economist Group, 13—CNH Industrial, 14—Harris Associates, 15—Giovanni Agnelli B.V., 16—Piero Ferrari, 17—T. Rowe Price Associates, Inc.; ---- company relationships relating to John Elkann; ….. company relationships relating to Andrea Agnelli; ---- company relationships relating to Alessandro Nasi; ---- company relationships relating to Nicolas Dufourcq (ND); ---- company relationships relating to Robert Peugeot (RP).

In the following, we studied a simplified real-world shareholding network, which we call the “Stellantis network”, with 17 companies (numbered from 1 to 17). It is represented as a directed graph in Figure 3 with the weights on the arcs representing the direct ownership of a firm in a company (in %). For clarity, the matrix *S* also presents the direct ownership relations for our example. We constructed the network and the matrix *S* using current sources (https://en.wikipedia.org/wiki/Stellantis, (accessed on 12 August 2021), https://www.marketscreener.com/quote/stock/STELLANTIS-N-V-117750959/company/ (accessed on 12 August 2021), https://en.wikipedia.org/wiki/Exor_(company) (accessed on 12 August 2021), https://www.exor.com/pages/investors-media/shareholders-corner/ownership-structure (accessed on 12 August 2021), https://corporate.ferrari.com/en/investors/stock-and-shareholder-corner/shareholders-structure (accessed on 12 August 2021), http://www.gedispa.it/it/governance/dati-e-informazioni-societarie.html, https://en.wikipedia.org/wiki/CNH_Industrial (accessed on 12 August 2021), https://en.wikipedia.org/wiki/Dongfeng_Motor_Corporation (accessed on 12 August 2021), https://money.cnn.com/quote/shareholders/shareholders.html?symb=BLK&subView=institutional (accessed on 12 August 2021), https://money.cnn.com/quote/shareholders/shareholders.html?symb=BLK&subView=institutional (accessed on 12 August 2021), https://media.peugeot-invest.com/2021-02/2021-02-22-changement-de-nom-ffp-uk.pdf (accessed on 12 August 2021), https://live.euronext.com/sites/default/files/cpr03_lesechos_16165_965485_2021_02_15_Changement_de_nom_Maillot_1__UK.pdf (accessed on 12 August 2021)).

In this automotive market—the Stellantis network—we found five people in more than one company leadership role.

John Elkann is Chairman and Chief Executive Officer of Exor N.V., Chairman of Stellantis N.V., Chairman of Ferrari N.V., Chairman of Giovanni Agnelli B.V., and Chairman of GEDI Gruppo Editoriale S.p.A. He is also a member of PartnerRe’s Board of Directors (https://www.exor.com/pages/exor/governance/board-directors, https://partnerre.com/contact/john-elkann (accessed on 12 August 2021)).

Andrea Agnelli is non-executive Director in Stellantis N.V and Exor N.V, a Director of Giovanni Agnelli B.V., and Chairman of Juventus Football Club S.p.A. (https://www.exor.com/pages/exor/governance/board-directors, https://www.juventus.com/en/club/corporate-governance/board/andrea-agnelli (accessed on 12 August 2021)).

Alessandro Nasi is a Director of CNH Industrial N.V. (from 2019), a Director of Giovanni Agnelli B.V., and Vice Chairman of the Board of Directors of Exor N.V. (https://www.cnhindustrial.com/en-us/governance/cnh_industrial_leaders/board_of_directors_documents/CV/CV_Nasi_ENG_June_22_2020.pdf, (accessed on 12 August 2021)).

Nicolas Dufourcq is non-executive Director of Stellantis. Nicolas Dufourcq has been the Chief Executive Officer of Bpifrance SA. He serves as Chairman and Chief Executive Officer of Bpifrance Participations S.A and he is a permanent representative of Bpifrance Participations S.A. on the board of directors of Orange (https://www.stellantis.com/en/group/governance/leadership, (accessed on 12 August 2021), https://www.stellantis.com/content/dam/stellantis-corporate/group/governance/leadership/bio/eng/Nicolas%20Duforcq.pdf (accessed on 12 August 2021)).

Robert Peugeot serves as non-executive Director of Stellantis and Vice Chairman. He is also Chairman of the board of Peugeot Invest (FFP S.A.), the company which, through the intermediary of subsidiary Peugeot 1810, owns a 7.16% stake in Stellantis (https://www.stellantis.com/en/group/governance/leadership, (accessed on 12 August 2021), https://live.euronext.com/sites/default/files/cpr03_lesechos_16165_965485_2021_02_15_Changement_de_nom_Maillot_1__UK.pdf (accessed on 12 August 2021)).

The values (in billion US dollars) of the companies in the Stellantis network are $\bar{W}$= (68.05, 20.53, 0.26, 3.36, 8.03, 6.39, 29.99, 139.72, 55.33, 0.16, 1.20, 0.002, 23.69, 0.00, 10.00, 0.00, 48.64), respectively. As most of the companies in the example are listed, the values in the vector W represent the stock exchange values, i.e., their market capitalization (Retrieved from https://companiesmarketcap.com/ (accessed on 12 August 2021)). The market capitalization is commonly used to measure the value of a publicly listed company. It is calculated by multiplying the current market price of a company’s shares with the number of outstanding shares (https://www.investopedia.com/investing/market-capitalization-defined (accessed on 12 August 2021)).

For company 4 (Peugeot family group), we consulted https://live.euronext.com/en/product/equities/FR0000064784-XPAR and used the market capitalization of Peugeot Invest. Peugeot Invest (formerly FFP) is a listed company which is one of the main shareholders of Stellantis.

For companies 10 (PartnerRe) and 11 (Juventus), we also retrieved the market capitalization values (https://www.google.com/search?q=market+apitalization+Partner+Re&oq=market+apitalization+Partner+Re&aqs=chrome..69i57j33i10i160.9683j1j15&sourceid=chrome&ie=UTF-8 (accessed on 12 August 2021), https://www.google.com/search?q=market+apitalization+Juventus+Football+Club&sxsrf=ALeKk03tdcNb5Is0ip7wptMEEpPlNA0mnQ%3A1628786295455&ei=d04VYeukG-rKrgTK57pI&oq=market+apitalization+Juventus+Football+Club&gs_lcp=Cgdnd3Mtd2l6EAMyBwgjELACECc6BwgAEEcQsAM6BAgAEBM6CAgAEBYQHhATSgQIQRgAUPTZB1iwmAhgwKIIaAFwAXgAgAF8iAG_BZIBAzQuM5gBAKABAaABAsgBCMABAQ&sclient=gws-wiz&ved=0ahUKEwirvLjk9avyAhVqpYsKHcqzDgkQ4dUDCA4&uact=5 (accessed on 12 August 2021)).

For an unlisted company such as company 3 (GEDI), we applied the last stock market/market capitalization (https://it.investing.com/equities/g-ed-lespresso (accessed on 12 August 2021)).

The value of company 6 (Bpifrance S.A.) was estimated with the help of share capital published in the UNIVERSAL REGISTRATION DOCUMENT 2020 (https://www2.bpifrance.fr (accessed on 12 August 2021)). Similarly, for company 12 (The Economist Group), we used its share capital published on page 11 of the Interim Report 2020 (https://www.economistgroup.com/pdfs/Interim_Report_2020.pdf (accessed on 12 August 2021)).

Companies 14 and 16 are not joint stock companies. Hence their values in the vector $\bar{W}$ are zero. Company 15 (Giovanni Agnelli B.V.) is a private company with share capital equal to Euro 50.000,00 (approximately 58.735 USD) (https://www.gazzettaufficiale.it/eli/id/2016/07/30/TX16AAB7340/p2 (accessed on 12 August 2021)). However, the Agnellis are worth more than $10 billion, according to the Bloomberg Billionaires Index (https://www.bnnbloomberg.ca/agnelli-fortune-gains-900-million-on-fiat-peugeot-mega-merger-1.1340757 (accessed on 12 August 2021)), thus we used this value in $\bar{W}$.

The individual shares (in %) of companies from the “Stellantis” market are presented in the matrix *S*.


$$S=\left[\begin{array}{ccccccccccccccccc} 100 & 0 & 0 & 0 & 0 & 0 & 0 & 0 & 0 & 0 & 0 & 0 & 0 & 0 & 0 & 0 & 0\\ 14.40 & 100 & 89.60 & 0 & 0 & 0 & 0 & 0 & 24.05 & 100 & 63.8 & 43.4 & 26.9 & 0 & 0 & 0 & 0\\ 0 & 0 & 100 & 0 & 0 & 0 & 0 & 0 & 0 & 0 & 0 & 0 & 0 & 0 & 0 & 0 & 0\\ 7.16 & 0 & 0 & 100 & 0 & 0 & 0 & 0 & 0 & 0 & 0 & 0 & 0 & 0 & 0 & 0 & 0\\ 4.91 & 0 & 0 & 0 & 100 & 0 & 0 & 0 & 0 & 0 & 0 & 0 & 0 & 0 & 0 & 0 & 0\\ 5.66 & 0 & 0 & 0 & 0 & 100 & 9.56 & 0 & 0 & 0 & 0 & 0 & 0 & 0 & 0 & 0 & 0\\ 0 & 0 & 0 & 0 & 0 & 0 & 100 & 0 & 0 & 0 & 0 & 0 & 0 & 0 & 0 & 0 & 0\\ 3.57 & 0 & 0 & 0 & 0 & 0 & 1.17 & 100 & 3.85 & 0 & 0 & 0 & 0 & 0 & 0 & 0 & 0\\ 0 & 0 & 0 & 0 & 0 & 0 & 0 & 0 & 100 & 0 & 0 & 0 & 0 & 0 & 0 & 0 & 0\\ 0 & 0 & 0 & 0 & 0 & 0 & 0 & 0 & 0 & 100 & 0 & 0 & 0 & 0 & 0 & 0 & 0\\ 0 & 0 & 0 & 0 & 0 & 0 & 0 & 0 & 0 & 0 & 100 & 0 & 0 & 0 & 0 & 0 & 0\\ 0 & 0 & 0 & 0 & 0 & 0 & 0 & 0 & 0 & 0 & 0 & 100 & 0 & 0 & 0 & 0 & 0\\ 0 & 0 & 0 & 0 & 0 & 0 & 0 & 0 & 0 & 0 & 0 & 0 & 100 & 0 & 0 & 0 & 0\\ 0 & 4.99 & 0 & 0 & 0 & 0 & 0 & 0 & 0 & 0 & 0 & 0 & 11.1 & 100 & 0 & 0 & 0\\ 0 & 52.99 & 0 & 0 & 0 & 0 & 0 & 0 & 0 & 0 & 0 & 0 & 0 & 0 & 100 & 0 & 0\\ 0 & 0 & 0 & 0 & 0 & 0 & 0 & 0 & 10.23 & 0 & 0 & 0 & 0 & 0 & 0 & 100 & 0\\ 0 & 0 & 0 & 0 & 0 & 0 & 0 & 0 & 4.33 & 0 & 0 & 0 & 0 & 0 & 0 & 0 & 100 \end{array}\right]$$

The following calculations were performed using the R programming language and the R package CoopGame [31,32]. Taking into account $\bar{W}$ and *S*, we calculated the values of the companies and their subsidiaries in the Stellantis network. The resulting values are presented in the matrix *W*.


$$W=\left[\begin{array}{ccccccccccccccccc} 68.05 & 0 & 0 & 0 & 0 & 0 & 0 & 0 & 0 & 0 & 0 & 0 & 0 & 0 & 0 & 0 & 0\\ 9.80 & 20.53 & 0.23 & 0 & 0 & 0 & 0 & 0 & 13.31 & 0.16 & 0.77 & 0.001 & 6.37 & 0 & 0 & 0 & 0\\ 0 & 0 & 0.26 & 0 & 0 & 0 & 0 & 0 & 0 & 0 & 0 & 0 & 0 & 0 & 0 & 0 & 0\\ 4.87 & 0 & 0 & 3.36 & 0 & 0 & 0 & 0 & 0 & 0 & 0 & 0 & 0 & 0 & 0 & 0 & 0\\ 3.34 & 0 & 0 & 0 & 8.03 & 0 & 0 & 0 & 0 & 0 & 0 & 0 & 0 & 0 & 0 & 0 & 0\\ 3.85 & 0 & 0 & 0 & 0 & 6.39 & 2.87 & 0 & 0 & 0 & 0 & 0 & 0 & 0 & 0 & 0 & 0\\ 0 & 0 & 0 & 0 & 0 & 0 & 29.99 & 0 & 0 & 0 & 0 & 0 & 0 & 0 & 0 & 0 & 0\\ 2.43 & 0 & 0 & 0 & 0 & 0 & 0.35 & 139.72 & 2.13 & 0 & 0 & 0 & 0 & 0 & 0 & 0 & 0\\ 0 & 0 & 0 & 0 & 0 & 0 & 0 & 0 & 55.33 & 0 & 0 & 0 & 0 & 0 & 0 & 0 & 0\\ 0 & 0 & 0 & 0 & 0 & 0 & 0 & 0 & 0 & 0.16 & 0 & 0 & 0 & 0 & 0 & 0 & 0\\ 0 & 0 & 0 & 0 & 0 & 0 & 0 & 0 & 0 & 0 & 1.2 & 0 & 0 & 0 & 0 & 0 & 0\\ 0 & 0 & 0 & 0 & 0 & 0 & 0 & 0 & 0 & 0 & 0 & 0.002 & 0 & 0 & 0 & 0 & 0\\ 0 & 0 & 0 & 0 & 0 & 0 & 0 & 0 & 0 & 0 & 0 & 0 & 23.69 & 0 & 0 & 0 & 0\\ 0 & 1.02 & 0 & 0 & 0 & 0 & 0 & 0 & 0 & 0 & 0 & 0 & 2.63 & 0 & 0 & 0 & 0\\ 0 & 10.88 & 0 & 0 & 0 & 0 & 0 & 0 & 0 & 0 & 0 & 0 & 0 & 0 & 10 & 0 & 0\\ 0 & 0 & 0 & 0 & 0 & 0 & 0 & 0 & 5.66 & 0 & 0 & 0 & 0 & 0 & 0 & 0 & 0\\ 0 & 0 & 0 & 0 & 0 & 0 & 0 & 0 & 2.40 & 0 & 0 & 0 & 0 & 0 & 0 & 0 & 48.64 \end{array}\right]$$

From the matrix *W* we obtain the following net values of the companies:
[−24.2918.73−0.234.873.346.72−3.224.91−23.49−0.16−0.77−0.001−9.003.6510.885.662.40]


Next, taking into account all permutations of the 17 companies, we calculated the contributions of the individual companies to the Stellantis market using Formula (1). As a result, we obtained the market value of each company $\bar{w}$ (*M*):
[45.1938.160.047.9511.1712.7126.96144.3433.220.010.480.00115.223.4420.245.3350.89]


Let us now consider the personal relations in the Stellantis network. There were five people in this market who we found in more than one company leadership board: John Elkann in companies 1, 2, 3, 9, 10, and 15; Andrea Agnelli in companies 1, 2, 11, and 15; Alessandro Nasi in companies 2, 13, and 15; Nicolas Dufourcq in companies 1, 6, and 7; and Robert Peugeot in companies 1 and 4.

Let us assume that companies with the same people form the so-called pre-coalitions. So, we have a new *M*′ market consisting of companies and their pre-coalitions: {1, 2, 3, 4, 5, 6, 7, 8, 9, 10, 11, 12, 13, 14, 15, 16, 17, {1, 2, 3, 9, 10, 15}, {1, 2, 11, 15}, {2, 13, 15}, {1, 6, 7}, {1, 4}}.

As in Example 4, suppose that the market values of each pre-coalition are equal to the sum of the market values resulting from the ownership relationships of each of the companies in that pre-coalition. For the *M*′ market, we obtain the following vector $\bar{w}$(*M*′) =
[45.1938.160.047.9511.1712.7126.96144.3433.220.010.480.00115.223.4420.245.3350.89136.86104.0773.6284.8653.13]

Then, we standardize the obtained values to the total value of this market amounting to 415.352. $\bar{w}$ (M′_stand_) =
[21.6218.260.023.805.356.0812.9069.0815.900.0050.230.0017.281.659.692.5524.3665.5049.8035.2340.6125.43]

As in Example 4, we assume that the final value of a given company consists of the “clean” (resulting directly from ownership relations) and “biased” parts (resulting from participation in the pre-coalition). For the market *M*′, we have *N*′ = 17 + 5 = 22, DF = *N*′ − 2 = 20, α = 0.05, *t*_critical_ = 2.086, and the lowest measure of personal relations and at the same time the shares of pre-coalition in each single company is equal to $r_{ij}^{p}$ = 0.42122.

Corresponding net values are ${\bar{w}}^{netto}=[w_{i}^{netto}]=$
[32.2338.8027.5610.661.0617.0010.291.5414.1427.5820.74−0.00029.011.7260.851.630.69−137.9462.94−29.68−34.21−10.71]

Using Formula (1) we obtain
[52.3955.3026.3313.986.3622.3122.7270.5429.4026.3320.020.000315.883.2967.774.1025.01-66.18-10.276.907.9515.20]

Equal division of the values of pre-coalitions among its companies leads to the following company values
[49.0544.0115.3021.586.3624.9625.3770.5418.3715.3017.450.000318.183.2956.474.1025.0]


Comparing the different values of companies in Table 7, we observe that

- Firms 4, 5, 6, 8, 14, 15,16 and 17 that only control other companies in the network, but are not controlled by any company, increased their market values (when the ownership structure is incorporated) as compared to their original values, i.e., their stock market values, see second and third columns in Table 7;
- Companies 1, 3, 7, 9, 10, 11, 12 and 13 that are only controlled by other firms in the network, but are not controlling any firms, see their market values (when ownership structure is incorporated) decreased as compared to their original values, i.e., their stock market values, see second and third columns in Table 7;
- All firms connected by personal relation structures, except companies 7 and 9, increased their market values in line with the valuation method used and the ownership structure along with the personal structure, see third and fourth columns in Table 7. Companies 7 and 9 are overestimated as their net values are negative (−3.22 and −23.49, respectively). So, their market values in line with the ownership relations decreased compared to their original stock exchange values, see columns 2 and 3 in Table 7. In our market with 22 entities (17 companies and 5 pre-coalitions), the market values of companies 7 and 9 are 22.72 and 29.40, respectively. Company 7 takes part in the pre-coalition {1, 6, 7}. The market value of precoalition {1, 6, 7} is 7.95. Thus, the fraction equal to 2.65 is not sufficient to increase the value of company 7 over the market value 26.96, see columns 3 and 4 in Table 7. Similarly, company 9 takes part in pre-coalition {1, 2, 3, 9, 10, 15}. The market value of this pre-coalition is negative and equals −66.18. The ratio assigned to company 9 is equal to $-\frac{66.18}{6}=-11.3$. Thus, the market value of company 9, based upon ownership and personal relations, decreases to 18.37 and is lower than its market value, in line with only the ownership structure.

## 7. Conclusions and Future Works

The estimation of a company’s value is an important issue in economics. The models presented in this paper are among the first to be based on the theory of cooperative games. We applied deductive reasoning, in the sense that we first derived our new models and then applied them to real-world data We are confident that our approach can be helpful in assessments of the true values of firms. In complex corporate structures, some companies hide their true values or conceal the ownership by registering their firms in tax havens, adopting Chinese box strategies, and so on. In consequence, it becomes challenging to assess their true (objective) values.

The paper presents a novel approach for evaluating the market value of a company based upon ownership using the generalized Shapley value [11]. We extended our model by incorporating personal relationships of the management. It is apparent that, apart from the already mentioned ownership and personal relations, there may be other, additional relations that may be considered important in a specific case. In such situations, the overlapping individual structures make the problem more complex still. However, we believe that the proposed solutions can be applied analogously in such cases.

In Section 5 and Section 6, we simply distributed the values of pre-coalitions equally among their member companies. In practice, an expert’s estimate could help reallocate these values in a more sophisticated manner. We hope that our new models will open the door for additional empirical research into company valuation based on ownership and personal relations.

In the paper we tested our model using a simplified real-world example of the automotive market—the “Stellantis network”, with 17 companies. The model confirmed the opinion of the Bloomberg Billionaires Index that Giovanni Agnelli B.V. (a private company with share capital equal to Euro 50,000) is worth much more than $10 billion (see Section 6 and Table 7). A component of our future research will be to elaborate a method for how the proposed model could be tested in real-world complex corporate structures, and to improve the method sufficiently to ensure the validity of results obtained.

A further development may include the application of the present approach to assess the value (importance) of arcs in market networks. The use of Shapley values to evaluate both elements (nodes and arcs) of a multigraph was undertaken in [33]. Approaches for the measurement of the indirect control power of firms and mutual connections in corporate shareholding structures have, for example, been proposed in [15,34,35]. We plan to extend these existing approaches [15,34,35] by evaluating arcs and mutual connections utilizing reliable software for solving cooperative games and computing power indices [31,32].

## Figures and Tables

**Table 1:** Contributions of individual companies to the market $M^{S}=\left\{a_{1},a_{2},a_{3},a_{4}\right\}$.

Permutation	Company 1 Contribution to the Structure	Company 2 Contribution to the Structure	Company 3 Contribution to the Structure	Company 4 Contribution to the Structure
**1-2-3-4**	50	47	13	9.5
**1-2-4-3**	50	47	13	9.5
**1-3-2-4**	50	47	13	9.5
**1-3-4-2**	50	47	13	9.5
**1-4-2-3**	50	47	13	9.5
**1-4-3-2**	50	47	13	9.5
**2-1-3-4**	60.5	25	13	9.5
**2-1-4-3**	60.5	25	13	9.5
**2-3-1-4**	60.5	25	13	9.5
**2-3-4-1**	60.5	25	13	9.5
**2-4-1-3**	60.5	25	13	9.5
**2-4-3-1**	60.5	25	13	9.5
**3-1-2-4**	60.5	47	25	9.5
**3-1-4-2**	60.5	47	25	9.5
**3-2-1-4**	60.5	47	25	9.5
**3-2-4-1**	60.5	47	25	9.5
**3-4-1-2**	60.5	47	25	9.5
**3-4-2-1**	60.5	47	25	9.5
**4-1-2-3**	60.5	47	13	30
**4-1-3-2**	60.5	47	13	30
**4-2-1-3**	60.5	47	13	30
**4-2-3-1**	60.5	47	13	30
**4-3-1-2**	60.5	47	13	30
**4-3-2-1**	60.5	47	13	30

**Table 2:** Matrices S and W for Example 4.

**Matrix**	**Coallition**	1	2	3	4	(1,3)	(1,4)	(2,4)
	1	1	0	0	0.35	0.75455	0.75455	0
	2	0	1	0.4	0.4	0	0	0.75455
	3	0	0	1	0.1	0.75455	0	0
S=	4	0	0	0.2	1	0	0.75455	0.75455
	(1,3)	0.75455	0	0.75455	0	1	0	0
	(1,4)	0.75455	0	0	0.75455	0	1	0
	(2,4)	0	0.75455	0	0.75455	0	0	1
	**Coallition**	1	2	3	4	(1,3)	(1,4)	(2,4)
	1	22.62782	0	0	2.00131575	21.79401474	21.38836866	0
	2	0	16.22556	2.5022556	2.287218	0	0	16.55755093
	3	0	0	6.255639	0.5718045	21.79401474	0	0
W=	4	0	0	1.2511278	5.718045	0	21.38836866	16.55755093
	(1,3)	17.07382158	0	4.720192407	0	28.88346	0	0
	(1,4)	17.07382158	0	0	4.314550855	0	28.34586	0
	(2,4)	17.07382158	12.2429963	0	4.314550855	0	0	21.94361

**Table 3:** Intuitionistic fuzzy sets for personal relationships in pre-coalitions.

Pre-Coalition	Company	Person	Intuitionistic Fuzzy Set for Personal Relationship		
			μ	v	π
(1,3)	Company 1	P2	0.7	0.25	0.05
	Company 3		0.3	0.3	0.4
(1,4)	Company 1	P1	0.5	0.3	0.2
	Company 4		0.8	0.1	0.1
(2,4)	Company 2	P3	0.3	0.6	0.1
	Company 4		0.9	0.1	0

**Table 4:** Measures of personal relationships for pre-coalitions.

Pre-Coalition	Correlation Coefficient
(1,3)	0.90375
(1,4)	0.65663
(2,4)	0.54325

**Table 5:** Matrices S and W for Example 5.

**Matrix**	**Coalition**	1	2	3	4	(1,3)	(1,4)	(2,4)
	1	1	0	0	0.35	0.858799	0.903029	0
	2	0	1	0.4	0.4	0	0	0.912498
	3	0	0	1	0.1	0.858799	0	0
S=	4	0	0	0.2	1	0	0.903029	0.912498
	(1,3)	0.858799	0	0.858799	0	1	0	0
	(1,4)	0.903029	0	0	0.903029	0	1	0
	(2,4)	0	0.9212498	0	0.912498	0	0	1
	**Coalition**	1	2	3	4	(1,3)	(1,4)	(2,4)
	1	22.62782	0	0	2.00132	24.80510	25.59712	0
	2	0	16.22556	2.50226	2.28722	0	0	20.02350
	3	0	0	6.25564	0.57180	24.80510	0	0
W=	4	0	0	1.25113	5.71805	0	25.59712	20.02350
	(1.3)	19.43276	0	5.37234	0	28.88346	0	0
	(1.4)	20.43357	0	0	5.16356	0	28.34586	0
	(2.4)	0	14.80579	0	5.21770	0	0.00000	21.94361

**Table 6:** Value of a given company (Example 4) when additional information about the market in terms of experts’ assessments is given.

	**Companies**			
	1	2	3	4
Value in line with the valuation method used	50.000	25.000	25.000	30.000
Value in line with the valuation method used and the ownership structure	57.875	41.500	16.000	14.625
Value in line with the valuation method used and the ownership structure along with the personnel structure	42.195	24.905	23.265	36.635
Value in line with the valuation method used and the ownership structure along with the personnel structure (fuzzy attempt)	23.351	23.984	35.116	47.550

**Table 7:** Values of companies in the Stellantis network when different information about the market is given.

Company	Stock Exchange Values (Billions of USD)	Value in Line with the Valuation Method Used and the Ownership Structure *W*(*M*)	Value in Line with the Valuation Method Used and the Ownership Structure along with the Personal Structure *W*(*M^P^*)
1 (Stellantis)	68.05	45.19	49.05
2 (EXOR N.V.)	20.53	38.16	44.01
3 (GEDI)	0.26	0.04	15.30
4 (Peugeot family group)	3.36	7.95	21.58
5 (Dongfeng Motor Corporation)	8.03	11.17	6.36
6 (Bpifrance)	6.39	12.71	24.96
7 (Orange)	29.99	26.96	25.37
8 (BlackRock)	139.72	144.34	70.54
9 (Ferrari)	55.33	33.22	18.37
10 (PartnerRe)	0.16	0.01	15.30
11 (Juventus Football Club)	1.20	0.48	17.45
12 (The Economist Group)	0.002	0.001	0.0003
13 (CNH Industrial)	23.69	15.22	18.18
14 (Harris)	0.00	3.44	3.29
15 (Giovanni Agnelli)	10.00	20.24	56.47
16 (Piero Ferrari)	0.00	5.33	4.10
17 (T. Rowe Price)	48.64	50.89	25.01

## Data Availability

Not applicable.
